# RETRACTION: “Knockdown of Zebrafish Blood Vessel Epicardial Substance Results in Incomplete Retinal Lamination”

**DOI:** 10.1155/tswj/9768631

**Published:** 2026-08-03

**Authors:** The Scientific World Journal

RETRACTION: Y‐C. Wu, R‐F. Chen, C‐Y. Liu, F. R. Hu, C‐J. Huang, and I‐J. Wang, “Knockdown of Zebrafish Blood Vessel Epicardial Substance Results in Incomplete Retinal Lamination,” *The Scientific World Journal,* no. 2014 (2014). 10.1155/2014/803718.

The above article, published online on 6 March 2014 in Wiley Online Library (wileyonlinelibrary.com), has been retracted following the agreement of the journal’s Academic Editor, Bhishma Karki; and John Wiley & Sons Ltd. The retraction has been agreed following an investigation into concerns initially raised by *Xenograpsus ngatama* on PubPeer [1]. The investigation identified issues related to Figure 4, in which panels (j) and (k) contained overlapping features despite representing different experimental conditions.

Subsequent investigation by the publisher identified further inconsistencies in the raw data provided by the authors. Specifically, an apparent duplication was found between a portion of Figure 1(i’) and Figure 2(k), although rotated and at different magnification, when the experiments described are under different conditions. In light of the above, there are significant concerns with the reliability of the information presented in the article, and therefore the parties agree that the article must be retracted.

The authors disagree with this retraction.
